# Dysphagia Lusoria in Childhood: An Uncommon Cause of Swallowing Difficulty

**DOI:** 10.7759/cureus.106779

**Published:** 2026-04-10

**Authors:** Ghassan A Sukkar, Halah Fallata, Rahaf Alharbi, Salma H Almadani

**Affiliations:** 1 Pediatric Gastroenterology, King Saud Bin Abdulaziz University for Health Sciences, Jeddah, SAU; 2 Pediatric Gastroenterology, King Abdullah International Medical Research Center, Jeddah, SAU; 3 Pediatric Gastroenterology, Ministry of the National Guard - Health Affairs, Jeddah, SAU; 4 Pediatrics, College of Medicine, King Saud bin Abdulaziz University for Health Sciences, Jeddah, SAU; 5 Pediatric Gastroenterology, King Abdullah Specialized Children Hospital (KASCH), Jeddah, Jeddah, SAU

**Keywords:** aberrant right subclavian artery, congenital aortic arch anomaly, feeding intolerance, pediatric esophageal compression, vascular ring

## Abstract

Dysphagia lusoria is a rare cause of esophageal compression, typically resulting from an aberrant right subclavian artery (ARSA). ARSA is the most frequently occurring anomaly of the aortic arch. Although usually asymptomatic, most patients complain of dysphagia or respiratory symptoms. This case is of a nine-year-old boy with asthma and allergic rhinitis who presented with chronic epigastric pain, vomiting, and progressive dysphagia to solids. His growth parameters were normal but on the lower end of the body mass index (BMI) range. Esophagogastroduodenoscopy showed a pulsatile external compression in the proximal to mid-esophagus. A retro-esophageal ARSA arising distally from the aortic arch is identified on a CT angiography. A multidisciplinary team advised surgical repair, and the patient underwent division and re-implantation of the ARSA. The postoperative course was uneventful, with marked improvement in dysphagia and vomiting. Early recognition and evaluation of dysphagia in children help minimize complications, leading to better outcomes.

## Introduction

Dysphagia lusoria is a rare medical condition characterized by external compression of the esophagus, leading to difficulty in swallowing, most commonly due to an aberrant right subclavian artery (ARSA), also known as arteria lusoria. ARSA represents the most frequent congenital anomaly of the aortic arch, with an estimated prevalence ranging from 0.16% to 4.4% in the general population [1].

Pediatric dysphagia is more commonly attributed to conditions such as gastroesophageal reflux disease, eosinophilic esophagitis, structural abnormalities, or esophageal motility disorders. However, vascular anomalies remain an important yet often overlooked cause.

First described by Hunauld in 1735 and later associated with dysphagia by Bayford in 1794, dysphagia lusoria typically arises when the aberrant artery follows a retroesophageal course [2,3]. In rare cases, it may pass between the trachea and esophagus or anterior to the trachea [3]. Although most individuals with ARSA remain asymptomatic, approximately 20%-40% develop symptoms, most commonly dysphagia or respiratory complaints [4]. In younger individuals and infants, symptoms can vary, often presenting as respiratory problems like stridor or frequent infections. [5] The onset of symptoms is thought to be influenced by several factors, including concomitant vascular abnormalities, age-related vascular rigidity, or aneurysmal dilatation [4].

## Case presentation

A nine-year-old male with a history of asthma and allergic rhinitis presented to the pediatric gastroenterology clinic with chronic epigastric pain occasionally requiring acetaminophen, not awakening him from sleep, and associated with daily non-bilious vomiting unrelated to oral intake. He also reported difficulty swallowing solid food and preferred soft, pureed food due to dysphagia not associated with food impaction. His growth parameters are within the normal range for age and sex, with BMI on the lower end of normal. He was born preterm with a low birth weight of 1.5 kg and had a history of frequent vomiting and gastroesophageal reflux symptoms during infancy.

Given the patient’s symptoms, differential diagnoses initially included gastroesophageal reflux disease, eosinophilic esophagitis, and esophageal motility disorders. However, the progressive nature of dysphagia to solids raised concern for a structural or extrinsic compressive etiology, prompting further diagnostic evaluation.

**Figure 1:**
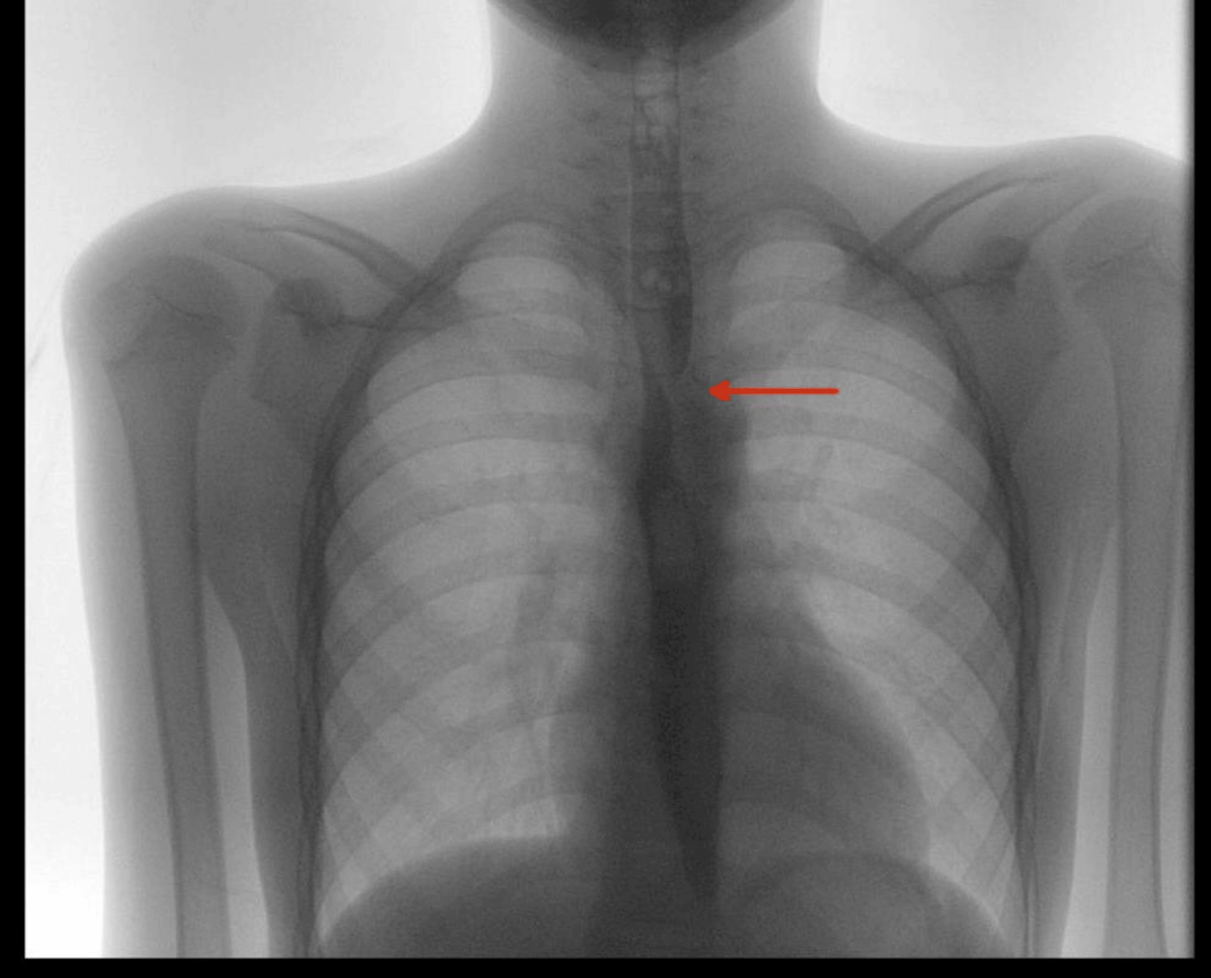
Upper gastrointestinal contrast imaging (normal esophageal caliber and mucosa (red arrow) and no evidence of filling defects, strictures, or diverticula).

**Figure 2:**
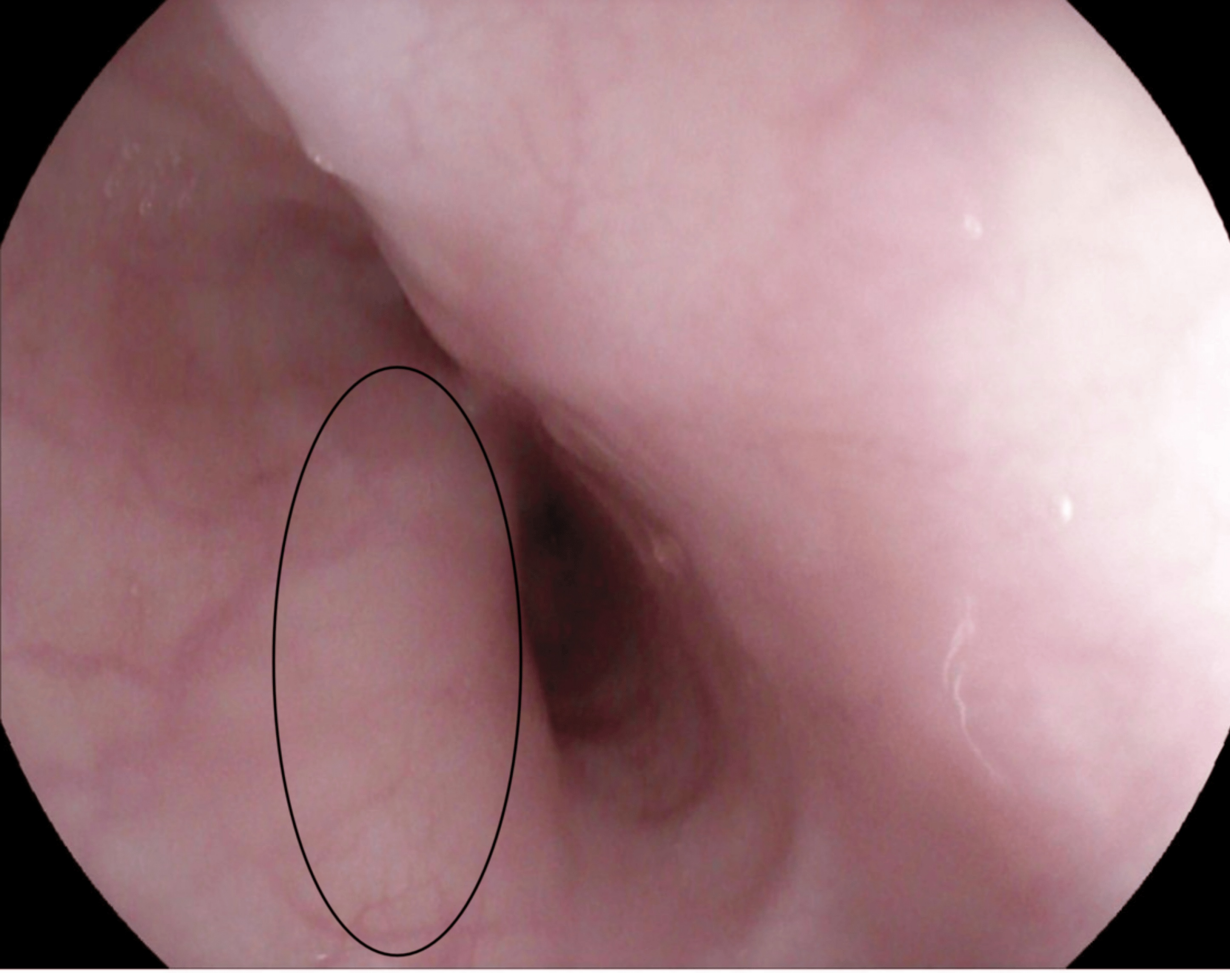
Esophagogastroduodenoscopy showing a pulsatile external compression of the mid-esophagus (outlined), consistent with vascular indentation.

**Figure 3:**
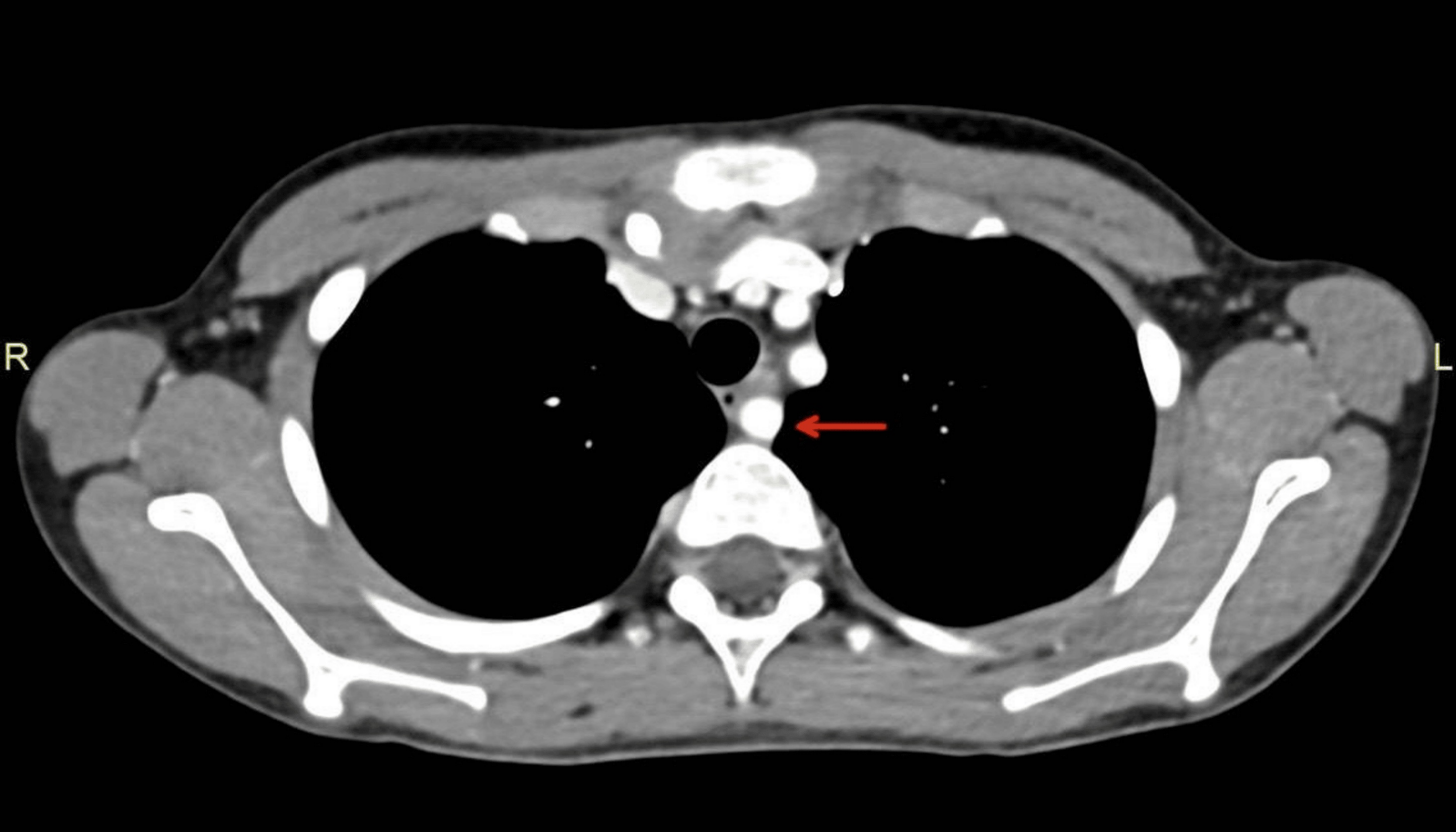
Cross-sectional view of CT angiography revealing an aberrant right subclavian artery (red arrow) coursing retroesophageally.

**Figure 4:**
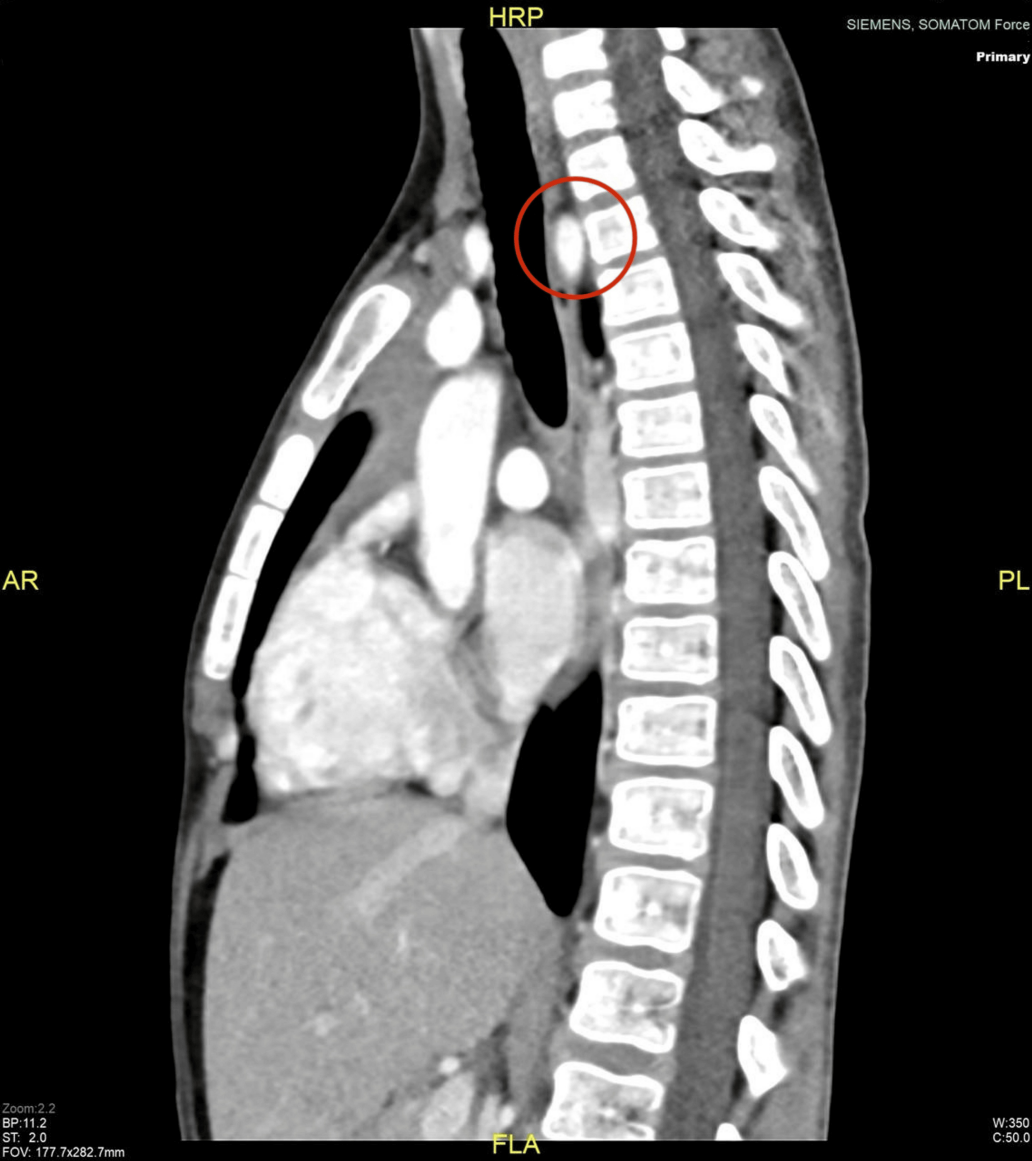
Sagittal view of CT angiography revealing an aberrant right subclavian artery (red circle) coursing retroesophageally.

An upper gastrointestinal (GI) barium study (Figure 1) demonstrated normal flow of contrast from the esophagus to the stomach, with normal esophageal caliber and mucosa and no evidence of filling defects, strictures, or diverticula. However, the study was suboptimal due to difficulty with contrast ingestion, limiting its diagnostic utility and necessitating further evaluation. Subsequently, esophagogastroduodenoscopy (EGD) revealed a pulsating external compression partially obstructing the lumen between the middle and proximal esophagus, with otherwise normal mucosa (Figure 2). Chest CT revealed a right aberrant subclavian artery arising from the arch of the descending thoracic aorta after the origin of the left subclavian artery, coursing retroesophageally (Figures 3-4). Echocardiography was normal for his age. A multidisciplinary team (MDT) discussion with cardiac surgery, gastroenterology, and vascular surgery concluded with a decision to proceed with surgical repair. The patient underwent vascular ring repair with Division and Re-implantation of the Right Aberrant Sub-clavian artery. The patient tolerated the procedure without complications. Following surgery, the patient’s symptoms improved markedly, with only occasional mild epigastric pain, and he continues to follow up with gastroenterology for nutritional optimization and growth monitoring.

## Discussion

Dysphagia lusoria refers to swallowing impairment caused by extrinsic compression of the esophagus by an ARSA. This anomaly is a developmental variant of the aortic arch where the right subclavian artery arises distally (often as the fourth branch) and traverses posterior to the esophagus to reach the right upper limb [3]. Similarly, anomalies could originate on the left and beyond the left subclavian artery, where it follows an oblique course, upward and on the right toward the right supraclavicular fossa [6]. ARSA has a female predominance reported as 63% of the cases [7]. Whereas most cases remain asymptomatic [4], clinical manifestation depends on several anatomical and age‐related factors [6]. Loss of rigidity of the trachea is thought to cause respiratory symptoms of ARSA, such as cough and dyspnea [6]. Though dysphagia is the most frequent complaint. Furthermore, weight loss may also occur [3,8]. Symptoms typically appear later in life when age-related factors such as arteriosclerosis, aneurysmal dilation (known as Kommerell’s diverticulum), or reduced esophageal compliance worsen the compression [4].

In this case, the patient presented with progressive dysphagia to solids, a characteristic feature of the condition. An ARSA is often identified as an incidental finding on imaging studies [7]. Diagnostic modalities include a barium swallow (esophagogram), which typically demonstrates external compression of the esophagus. On endoscopic ultrasound, ARSA appears as an anechoic tubular structure located between the esophagus and the spine, showing a positive Doppler signal, and has been reported as an incidental finding in all patients studied [7]. Additionally, upper endoscopy may reveal a pulsatile external indentation of the esophageal wall. However, cross-sectional imaging techniques such as CT angiography or MR angiography remain the definitive diagnostic tools, as they provide an accurate and detailed representation of the vascular anatomy [1,3]. In addition to endoscopy and cross-sectional imaging, esophageal manometry may provide useful information in selected cases by evaluating esophageal motility and excluding primary motility disorders. Endoscopic ultrasound can also assist in identifying vascular structures adjacent to the esophagus. These modalities may be particularly valuable in cases where initial imaging is inconclusive or when symptoms are disproportionate to structural findings [8]. In our case, the inconclusive barium study, combined with persistent symptoms, guided the decision to proceed with endoscopy and advanced imaging, ultimately leading to definitive diagnosis.

Management strategies are dictated by the severity of symptoms and the presence of any complications [3]. Individuals with no symptoms typically do not require any treatment. Mild symptoms can be addressed conservatively through dietary changes, proton pump inhibitors, and swallowing therapy [9,10]. On the other hand, patients experiencing severe or worsening symptoms may need surgical or endovascular procedures. Treatment options include the division and reimplantation of the ARSA into the carotid or subclavian artery, hybrid methods, or the exclusion of a Kommerell’s diverticulum. The selection of the procedure is tailored to the individual, considering comorbid conditions, vascular anatomy, and the expertise of the surgeon. [9] In our case, the patient underwent division and re-implantation of the right aberrant subclavian artery with anastomosis to the innominate artery. The procedure and recovery were uneventful, with stable postoperative outcomes. This case underscores the significance of recognizing vascular anomalies when diagnosing unexplained dysphagia, particularly when endoscopy yields no findings. Prompt identification using suitable imaging can help avoid unnecessary delays and guide optimal therapy [3]. Reporting such cases enhances our understanding of the diverse presentations and management results associated with dysphagia lusoria [9].

## Conclusions

Early recognition and systematic evaluation of pediatric dysphagia, with consideration of both common and rare etiologies, are essential to avoid delayed diagnosis. While more prevalent causes, such as reflux or eosinophilic esophagitis, should be excluded, clinicians should maintain a high index of suspicion for vascular anomalies in cases of persistent or unexplained symptoms.

In our case, timely diagnosis and surgical intervention resulted in significant clinical improvement with an uneventful postoperative course. This highlights the importance of a structured diagnostic approach and multidisciplinary management in optimizing patient outcomes. Long-term follow-up remains essential to ensure sustained symptom resolution and appropriate growth.
